# Alterations in ganglion cell and nerve fiber layer in Leber hereditary optic neuropathy across clinical stages

**DOI:** 10.1186/s12886-025-03991-3

**Published:** 2025-04-08

**Authors:** Michael Oeverhaus, Mareile Knetsch, Ying Chen, Leyla Jabbarli, Carmen Nolden, Anja Eckstein, Nikolaos E. Bechrakis, Philipp Rating

**Affiliations:** 1 Department of Ophthalmology, University Hospital Essen, Essen, Germany; 2 Medical Practice Dres. Oeverhaus, Rietberg, Germany

**Keywords:** LHON, SD-OCT, GCL, mtDNA mutation

## Abstract

**Purpose:**

LHON leads to gradual, painless, and permanent vision loss in both eyes, often associated with central scotomas. As the condition progresses, there is a decline in visual function, accompanied by noticeable structural alterations. This study focused on evaluating the clinical characteristics of patients with differing LHON stages, with a specific emphasis on optical coherence tomography (OCT) imaging results.

**Methods:**

This analysis included 22 individuals with LHON. Patients underwent thorough clinical ophthalmologic assessments, including SD-OCT, Visual evoked potentials, and perimetry. When LHON was suspected, blood samples were obtained to test for the three major mitochondrial mutations (G1178A, T14484C, G3460A), with further sequencing to identify additional known mutations. The data were subsequently examined through descriptive statistical methods.

**Results:**

The clinical characteristics of 22 individuals (median age 33, range 9–68) were examined. All participants carried a mutation linked to LHON. The most prevalent mutation was G11778A (55%), followed by G3460A (23%), T14484C (14%), with one instance each of the rare G13042A and C3461T mutations. Fourteen participants experienced acute vision loss (average duration: 5.2 ± 5 months), while eight had chronic LHON. There was no significant difference in visual acuity (VA, logMAR) between the two groups (0.9 vs. 0.9, *p* = 0.91). However, chronic patients exhibited significantly reduced the retinal nerve fiber layer (RNFL), especially in the temporal region (32 μm vs. 56 μm, *p* < 0.0001), but not in the nasal region. Ganglion cell layer (GCL) thickness was also notably thinner in the temporal area for chronic patients compared to those with acute LHON (22 μm vs. 28 μm, *p* = 0.04). Linear regression analysis showed correlations between RNFL and GCL and visual acuity (R² = 0.18, *p* = 0.007 and R² = 0.1, *p* = 0.05).

**Conclusion:**

In our analysis, we observed an unusual pattern in the genetic mutations, with G3460A being the second most frequent, rather than T14484C, which may be attributed to the limited sample size. 14 patients experienced acute or subacute vision loss, while eight were assessed for chronic disease. Those with chronic LHON demonstrated significantly thinner GCL and RNFL. These results underscore the importance of accelerating both diagnosis and treatment to facilitate prompt intervention for patients.

## Introduction

Leber Hereditary Optic Neuropathy (LHON) is a rare, mostly maternally inherited, mitochondrial disease that causes blindness and selectively targets retinal ganglion cells (RGCs), particularly those originating from small axons in the macula [1, 2]. The disease is primarily caused by mitochondrial dysfunction resulting from inherited mitochondrial DNA (mtDNA) point mutations affecting the electron transport chain [3–5]. Of these, three mutations at nucleotide positions m.11778G > A, m.3460G > A and m.11484T > C account for approximately 90% of LHON cases. Several factors may trigger the disease like nuclear genetic modifiers or environmental exposures such as tobacco and alcohol consumption [6, 7]. LHON has a presymptomatic stage characterized by typical clinical findings as swelling of the peripapillary retinal nerve fiber layer (RNFL) and microangiopathy, followed by an acute stage with a rapid decline in visual acuity and development of a central scotoma, both worsening over several weeks [8, 9]. The disease progresses to a chronic stage with a stable and profound visual loss and central scotoma [10]. Although some patients may experience modest recovery, the disease is generally irreversible [11]. A definitive diagnosis requires genetic testing to differentiate to other hereditary and exclusion of acquired inflammatory, infective, compressive, toxic, and nutritional causes of optic neuropathy. In Europe the only approved treatment is Idebenone, a synthetic short-chain benzoquinone that bypasses the dysfunctional complex I to restore ATP production. Thus, 46% of patients gain a clinically relevant increase in visual acuity [12]. Gene therapy has been propsoed but is still under investigation [12–15].

Optical coherence tomography (OCT) has enabled clinicians and researchers a better understanding of LHON, particularly in the analysis of the thickness of the peripapillary RNFL and the RGC and inner plexiform layer (GC-IPL) using automated algorithms. The disease affects the RNFL thickness in a specific pattern, with early swelling in the superior and inferior quadrant followed by nasal swelling. In the chronic stage late thinning with preservation of the nasal quadrant has been observed [8, 16]. The RNFL swelling is likely due to compensatory increases in mitochondrial biogenesis and/or axonal stasis, which does not allow the detection of optic nerve atrophy in the early stages of the disease. This seems to be possible with measurements of the GC-IPL, where the influence of retinal vessels on measurements can be avoided. Here, even 6 weeks before onset of the symptoms specific patterned loss has been observed, starting with nasal sectors and progressing to the inferior sectors, which is reflecting the anatomical course of the papillomacular fibres [17]. All RNFL and GCL changes are accompanied by vascular changes in form of choroidal thickening [18]. We aimed to analyze the patients of our tertiary referral center with SD-OCT to better understand the differences between acute and chronic patients and the relationship of morphological and functional changes.

## Patients and methods

### Study population

This retrospective study analyzed the medical records of patients diagnosed with Leber Hereditary Optic Neuropathy (LHON) who attended our tertiary referral center between January 2012 and December 2022. Baseline demographic and clinical characteristics, as well as the disease course, were assessed. Clinical stages were classified by time from onset of the disease until data collection. Patients with clinical signs like vision loss from 0 to 12 months were assessed as acute and those with symptoms starting more than 12 months ago as chronic cases. Only treatment naïve patients with complete datasets were included in the analysis.

### Clinical assessment

**Fig. 1:**
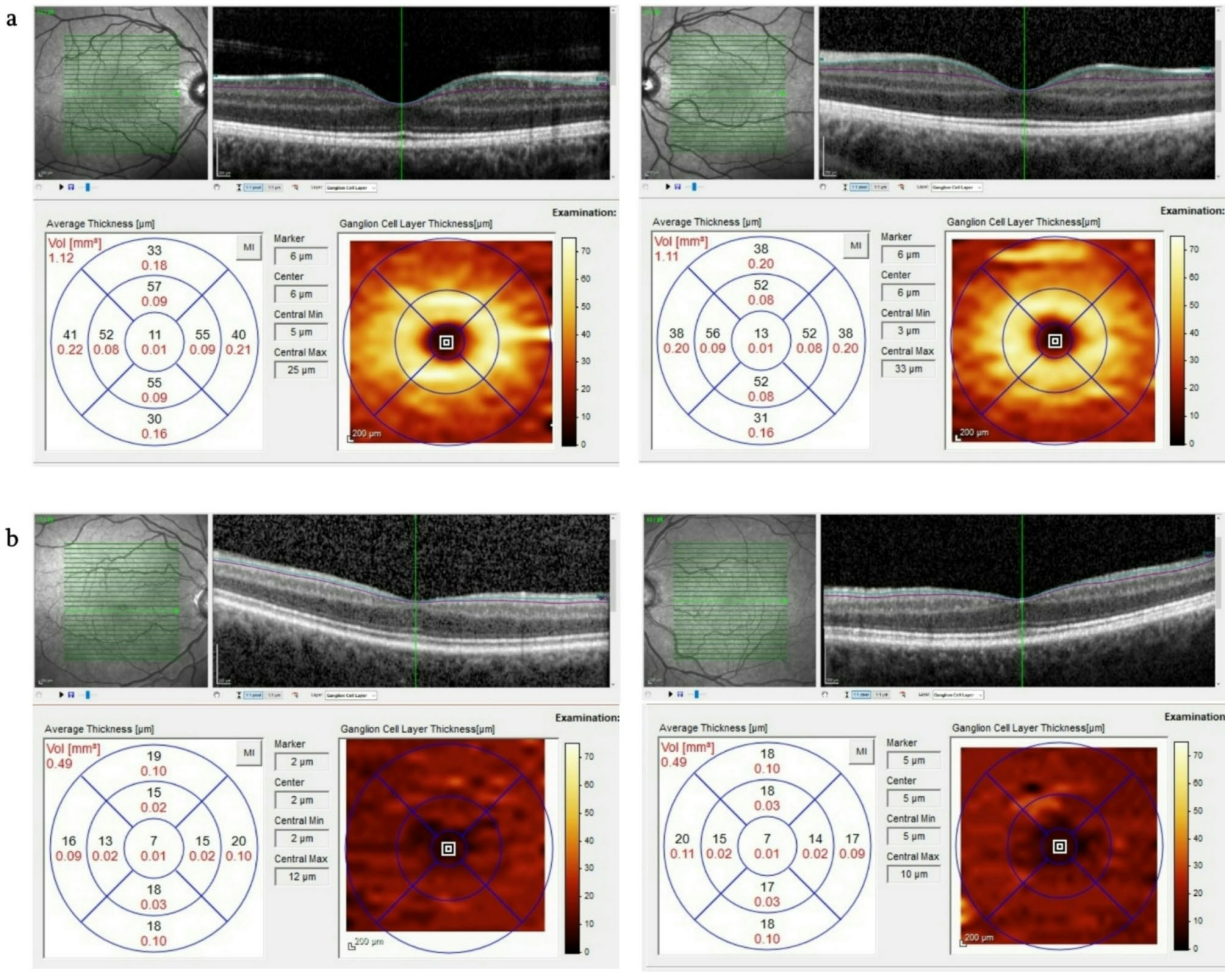
Cross-sectional OCT images and Ganglion Cell Layer maps (right and left eye) of a patient in an acute stage **(a)** and in a chronic stage **(b)** of LHON

All patients were evaluated by a multidisciplinary team of specialized ophthalmologists (MK, PR, MO, LJ, CN, YC). The diagnosis of LHON was established based on characteristic clinical features identified during comprehensive ophthalmologic examinations in our department, which included: BCVA, slit-lamp biomicroscopy, applanation tonometry, funduscopy, SD-OCT, VEP and perimetry. No patient showed a high myopia (>-5dpt) measured by auto refraction meter. Visual acuity (VA) was assessed at 5 m distance with the patients best refractive correction assessed by subjective refraction. The tests were performed using numbers as optotypes following routine protocol in the outpatients clinic. If patients were unable to identify any numbers, the distance was reduced to 1 m (visual acuity chart). If still no numbers were correctly identified, the VA was recorded as the ability to count fingers, recognize hand movements, perception of light, or absence of light perception. VA was recorded as decimal VA and afterwards converted to logMAR. ‘Counting fingers’ and ‘Hand motion’ were quantified as 1.9 and 2.3 logMAR, respectively, as previously published [19, 20]. SD-OCT (Spectralis^®^ OCT, Heidelberg Engineering, Heidelberg, Germany, Software Version: 6.15.7) was conducted by technicians in a special unit after standardized protocols of the manufacturer. Infrared (IR) and OCT images were simultaneously obtained with gaze tracking enabled (OCT: 768 × 496 Automatic Real Time (ART) with 100 images, IR: 768 × 786, ART 40 images). Retinal nerve fibre layer (RNFL) thickness was measured by IR&OCT 30° ART peripapillary retina CC7.7 (BL) program dedicated to optic nerve (Nsite). It was determined automatically after classification for six different sectors (nasal superior, nasal, nasal inferior, temporal inferior, temporal, temporal superior). Ganglion cell layer (GCL) thickness was automatically calculated and documented for the centre and 3 mm ETDRS circle in quadrants (superior, nasal, inferior and temporal). All automatic thickness measurements were corrected by refinement of margin lines (Fig. 1). The VEP (Reti-port/scan21, Roland Consult, Brandenburg, Germany, Software Version: 1021.3.0.0) electrophysiological assessment was carried out by technicians in accordance to standards of the International Society for Clinical Electrophysiology of Vision (ISCEV) in a special unit. Determination of the visual field was realized with Goldmann perimeter (Haag-Streit, Bern, Switzerland) as kinetic perimetry with stimulus size III and I (intensity 2–4) or with 30° Tubingen automated perimetry, TAP; Oculus Twinfield, Oculus, Wetzlar, Germany). If LHON was suspected, 7.5 ml EDTA blood samples were collected to screen for three main mitochondrial mutations (G1178A, G3460A, T14448C) and subsequently for further known mutations with Next Generation Sequencing. All clinical assessments and measurements were taken out before any therapeutic interventions such as idebenone.

### Statistical evaluation

For the analysis of metric data, median values ($\widetilde x$) with ranges or means with standard deviations (SD ±) were calculated, depending on the data distribution. Group comparisons were performed using a two-tailed Student’s *t*-test if normality was confirmed by the D’Agostino-Pearson omnibus normality test; otherwise, the Mann-Whitney *U* test was applied. Fisher’s exact test was employed to assess differences in the distribution of binary variables between groups. To identify independent factors associated with vision loss, multivariable logistic regression analysis was conducted. Significant variables considered for inclusion were temporal RNFL thickness, age at onset, duration since onset, male sex, and the G1178A mutation. Statistical significance was defined as two-tailed, with a threshold of α < 0.05. All statistical analyses were performed using SPSS (IBM SPSS Statistics, Version 22.0.0, Chicago, IL, USA) and GraphPad Prism (Version 9.0.0, GraphPad Software, San Diego, CA, USA). *P*-values are reported descriptively without adjustments for multiple testing.

## Results

### Study population

The study included a total of 22 patients with LHON (median age 33 years, range 10–67). Group 1 was composed of 14 patients (10 males, 4 females) with an acute course of the disease (median age 26, range 10–67). Group 2 comprises 8 patients (7males, 1 female) with chronic LHON (median age 34, range 12–68). Table 1 summarizes the demographics of the study population. Whereas the age of first visit as shown above was not significantly different between the two groups (*p* = 0.81), the age at onset was significantly different between acute and chronic LHON (26 yrs [10–67]; 34 yrs [12–68] *p* = 0.009). Family history of LHON was present in 41% (*n* = 9) of all patients, 5 in of those with acute LHON and 4 in chronic LHON patients without significance between the groups (*p* = 0.662). All 22 patients had a positive test for a LHON associated mutation. The G11778A mutation was the most common among the patients (55%), followed by G3460A (23%), T14484C (14%) and one rare G13042A mutation and one C3461T mutation. All patients were prescribed with Idebenone, either at their first visit (5 patients) or after receiving the genetic results (17 patients, mean 3.9 months until prescription).

**Table 1:** Characteristics of study population

	All (*n* = 22)	Acute (*n* = 14)	Chronic (*n* = 8)	*p*
Age at first visit (years)	33 [9–68]	26 [10–67]	34 [12–68]	0.81^a^
Age at onset (years)	19 [2–67]	26 [9–67]	15 [2–23]	0.009^a^
Male sex	77% (17)	71% (10)	88% (7)	0.613
Family history present	41% (9)	36% (5)	50% (4)	0.662
Visual acuity better eye (logMAR)	0.9 ± 0.5	0.9 ± 0.5	0.9 ± 0.7	0.91^a^
Visual acuity worse eye (logMAR)	1.3 ± 0.4	1.2 ± 0.4	1.5 ± 0.4	0.09^a^
Type of Mutation				
G11778A/ND4	55%	57%	50%	1.0^b^
T14484C/ND6	14%	14%	13%	1.0^b^
G3460A/ND1	23%	14%	38%	0.3089^b^
Time till first visit (months)	6.6 [0.5–571]	5.5 [0.5–16]	245 [25–571]	< 0.0001^a^
Time till genetics (months)	0.95 [0.2-9]	0.6 [0.2-7]	3.5 [0.2-9]	0.48^a^
Time till treatment (months)	11 [0.1–575]	7 [0.1–59]	250 [28–575]	< 0.0001^a^
VEP pathological	10/10	6/6	4/4	1.0
Central scotoma	18/18	11/11	7/7	1.0

Unless otherwise stated data are means ± SD or proportions (%) or median ($\widetilde x$) [range]; a: t-test/ Mann-Whitney-test both adjusted for multiple testing, b: Fishers exact test

### Clinical assessment

The assessment was adjusted for patients with optic neuropathy and performed as in clinical routine. We focused on the relevant general tests of individuals. Visual acuity and visual fields, on clinical examination of the fundus and visual evoked potential (VEP). The mean VA of the collective was 0.9 ± 0.5 logMAR in the better eye and 1.3 ± 0.4 logMAR in the worse eye (see Table 1). The VA of the patients better eye in Group 1 was 0.9 ± 0.5 logMAR and 0.9 ± 0.7 logMAR in Group 2, which is not significant (*p* = 0.91). We also revealed no difference in VA between the acute and chronic group for the worse eye (1.2 ± 0.4 vs. 1.5 ± 0.4; *p* = 0.09). The optic disc of all patients was assessed and classified by physicians through indirect funduscopy of both eyes. Typical pathological findings were present in 77% of all eyes (*n* = 44). Of these, 16 eyes showed a temporal and 16 eyes a total paleness. One patient in an acute situation had bilaterally blurred edges of the optic disc in both eyes. Ten eyes had no fundoscopic signs of any optic nerve pathology. Visual field testing with Goldman perimeter or TAP revealed in all tested patients 18/22 pathologies (11 acute, 7 chronic). 13 (72%) of the tested showed central scotoma and 2 (11%) an increased blind spot in both eyes. One patient showed a unilateral scotoma, another a unilateral increased blind spot, and one a mixed clinic. The patient with the mixed clinic was from the acute group and had an onset of symptoms just 3 weeks before examination. Visual evoked potentials (VEP) were conducted in 10 (43%) of all 22 patients. All 10 (6 acute, 4 chronic) examined patients showed a P100 amplitude reduction and two of them an additional P100 latency delay.

### OCT Analysis

**Fig. 2:**
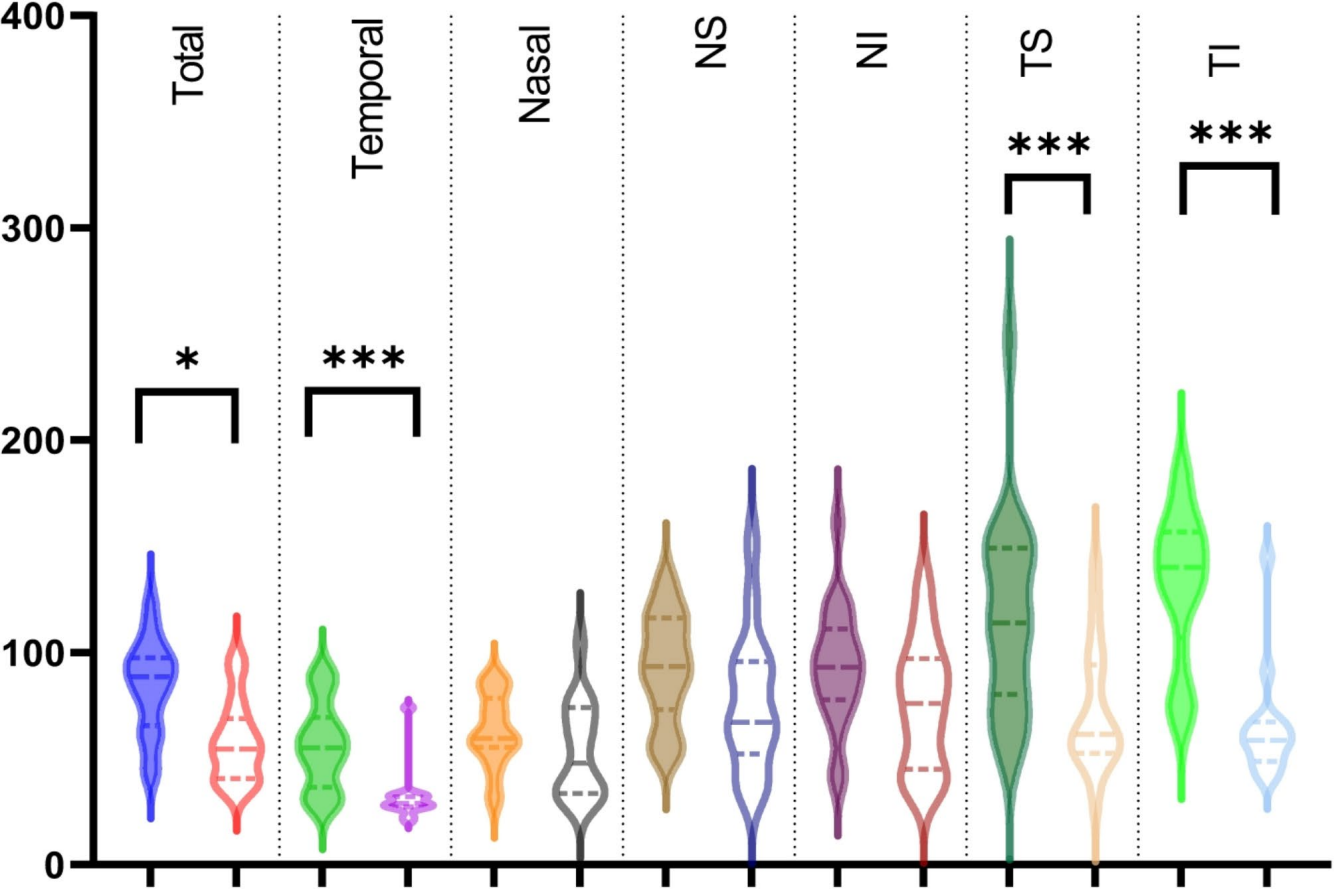
Violin plots comparing the RNFL thickness of acute (filled plots) and chronic (unfilled plots) LHON patients dependent on the topography (NS: nasally superior, NI: nasally inferior, TS: temporally superior, TI: temporally inferior)

Mean RNFL (Retinal nerve fiber layer) thickness was significantly lower in chronic patients (58 μm vs. 84), especially temporally (32 μm vs. 56, *p* < 0.0001). Table 2; Fig. 2 show the mean RNFL thicknesses of all six sectors. The mean thickness temporal superior was also significantly lower in chronic compared to acute patients (73 μm vs. 117 μm, *p* < 0.0001). The same was true for the temporal inferior sector (64 μm ± 25 vs. 132 μm ± 36, *p* < 0.0001). In contrast nasal sectors showed no significant differences between the groups.

**Fig. 3:**
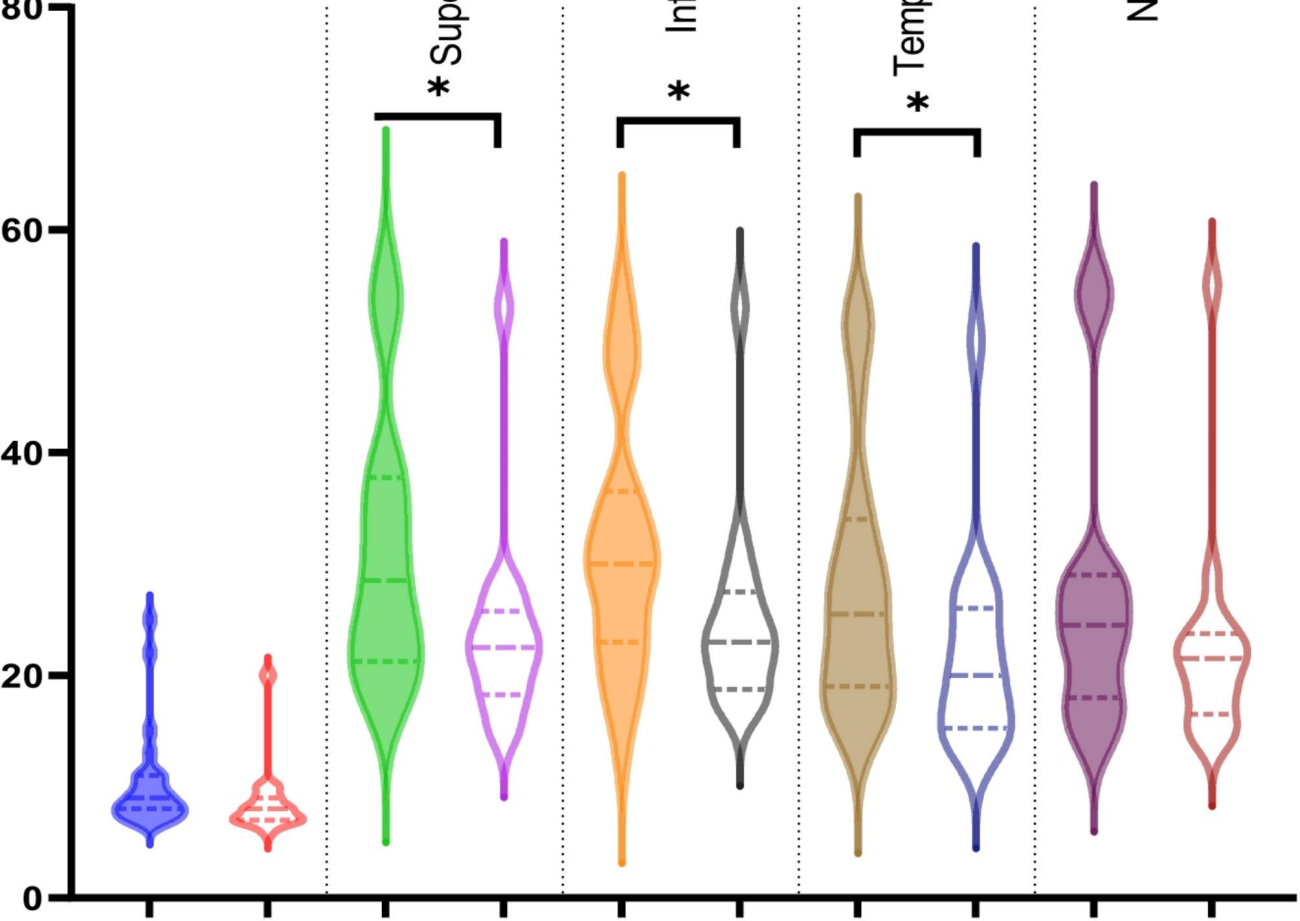
Violin plots comparing the GCL thickness of acute (filled plots) and chronic (unfilled plots) LHON patients dependent on the topography (EDTRS grid)

The foveal Ganglion cell layer (GCL) thickness was not significantly reduced (*p* = 0.07, see Fig. 3). Still, there was a significant reduction in the temporal (*p* = 0.05), superior (*p* = 0.03) and inferior (*p* = 0.02) sectors in chronic patients compared to acute patients (see Table 2). Nasally there was no statistical significant difference. Please note, that two patients did not receive a RNFL but a GCL measurement and two patients a RNFL but not a GCL scan, resulting in 40 eyes for OCT analysis in both aspects.

**Table 2:** OCT layer analysis

	All	Acute	Chronic	*p*
RNFL total (all in µm)	70 ± 25	84 ± 22	58 ± 19	**0.04**
temporal	43 ± 21	56 ± 20	32 ± 12	**< 0.0001**
temporal superior	94 ± 44	117 ± 46	73 ± 29	**< 0.0001**
temporal inferior	97 ± 46	132 ± 36	64 ± 25	**< 0.0001**
nasal	57 ± 20	63 ± 17	54 ± 23	0.98
nasal superior	83 ± 30	93 ± 26	76 ± 32	0.61
nasal inferior	83 ± 30	93 ± 28	76 ± 30	0.50
GCL Fovea (all in µm)	9.6 ± 4	10 ± 5	9 ± 3	0.07
superior	28 ± 12	32 ± 12	24 ± 9	**0.03**
inferior	29 ± 11	32 ± 11	25 ± 9	**0.02**
temporal	26 ± 11	28 ± 12	22 ± 9	**0.04**
nasal	26 ± 12	28 ± 13	22 ± 10	0.12

All values in µm. Unless otherwise stated data are means ± SD; *P*-Values for ANOVA-Model adjusted for multiple comparison

### Multiple linear regression

**Fig. 4:**
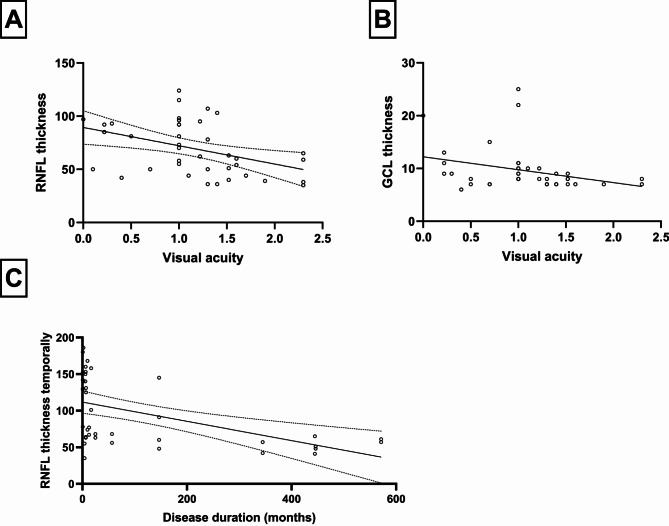
Linear regression depicting the association of visual acuity (VA) and RNFL thickness **(A)** and GCL thickness **(B)**. **(C)** depicts the association between the temporal RNFL thickness and the duration of the disease. The dotted lines depict the 95% confidence interval of best fit line and the unfilled dots the data points. Linear regression revealed significant RNFL and GCL thickness association with the corresponding VA (R²=0.18, *p* = 0.007 and R²=0.1, *p* = 0.05) and a significant correlation between RNFL thickness temporally and duration of the disease (R²= 0.26, *p* = 0.0007)

To check if the morphological parameters analyzed with OCT are associated with the visual function we performed a linear regression. Linear regression revealed significant RNFL and GCL thickness association with the corresponding VA (R²=0.18, *p* = 0.007 and R²=0.1, *p* = 0.05, see Fig. 4). Here, R² were 0.18 and 0.1, indicating that only 18% and 10% of the variance of VA could be explained by the models. Therefore, we performed a multiple linear regression to analyze further factors possibly associated with VA. The model incorporated besides temporal RNFL thickness, age at onset, time since onset, sex and presence of G1178A mutation. It showed that only RNFL thickness was significantly associated with VA (F [1;32] = 6.550; *p* = 0.02). Age showed a trend (F [1, 32] = 3.002; *p* = 0.09), but the other factors did not contribute to the model: Time since onset (*p* = 0.95), sex (*p* = 0.16), G1178A mutation (*p* = 0.12). Multicollinearity analysis was employed to ensure the independence of the five variables, which was the case. Goodness-of fit analysis showed a R² of 0.35 indicating that still only 35% of variance could be explained by the model.

Furthermore, we analyzed if the RNFL thickness showed a clear association with the duration of the disease. Since the group comparison revealed the most significant differences temporally, we chose this sector for the linear regression. It showed a significant correlation between RNFL and duration of the disease (*p* = 0.0007, s. Figure 4C). However, R² was only 0.26, indication further influential factors.

## Discussion

Our 10 year retrospective tertiary centre study reveals significant morphological SD-OCT differences in RNFL and GCL between the acute and chronic state of LHON. These results are in agreement with previous studies evaluating macular thickness differences in SD-OCT.

### Study cohort

The prevalence rate of the rare LHON is 1:54,000 based on a 70 year retrospective Danish national register analysis (male 1:34,000, female 1:123,000) including genetic testing [21]. The small cohort of our tertiary center collective (*n* = 22) reflects this low prevalence of this rare disease. Recent literature describes a 3:1 male-to-female ratio in LHON patients [22] which is also the case in our cohort (male sex 77%), which is unlike the traditional 5:1 male-to-female ratio commonly reported [12, 21, 23]. Mostly symptoms start in adolescence between the second and third decade of life. The age at onset was distributed over 6.5 decades with a median value of 19 years in our group which underlines a need for early diagnostic and imaging procedures in young adults. A long period of time (6 months in acute and 20 years in chronic patients) between the above mentioned age of onset and first visit in our ambulance speaks for the need of much work on public enlightment for this rare hereditary eye disease in our region. The distribution of the three main causal mutations in our analysis differs from previously published. We found the G3460A/ND1 (23%) mutation was more common than the T14484C/ND6 (14%) mutation [21, 24]. Poincenaut et al. (2020) harbored out in a recent international study with a large collective (*n* = 1512) of individuals with a known pathogenic LHON mutation that the second most frequent m.14,484 (17%) was only slightly larger than the m.3460 (13%) group [22]. This data supports the assumption that the distribution of LHON mutations in our collective is due to the rather small sample size. However, the most common G11778A/ND4 mutation is responsible up to ¾ LHON cases worldwide and in more than a half of our study population [25]. Interestingly, we found despite the small sample size rare genetic mutations, one G13042A and one C3461T mutation, which underlines the value of Next Generation Sequencing (NGS) in comparison to only perform Sanger Sequencing of the three most common mutations [26]. Besides the genetic testing the high rate of positive family history revealed the importance of genealogical trees. Despite the nowadays smaller family sizes the family history should always be inquired since it might give important clues for the differential diagnosis in cases with vision loss. Sporadic LHON cases are rare [27]. Our patients showed similar reduction of visual acuity as in prior studies [26]. Objective functional examinations with VEP derivation had a 100% pathological outcome which is highly reflected in subjective visual field testing of our patients and might be an indicator for their good test compliance [24]. 

### OCT Analysis

The analysis of the SD-OCT scans of the 14 patients with acute and 8 patients with chronic LHON revealed significant differences. Whereas, the early phase of the disease is characterized even by a thickening of the RNFL layer compared to a normal population (clinically swollen disc), the OCT of the chronic patients showed a significantly thinner nerve fiber layer [8, 28]. This was not only observed in the total RNFL thickness, but even more pronounced in the temporal sectors. In contrast, nasal sectors were not significantly thinner in chronic patients compared to acute patients. This is probably due to the fact, that the first signs of this atrophy, although not significantly, can already be found at 3 months after disease onset as a small longitudinal study showed [8]. At the 9 months follow-up it was also significant in all but the nasal quadrants. To further analyze the association between RNFL thickness temporally and disease duration our linear regression revealed a significant correlation. The high variability at the onset of the disease (s. Figure 4C) shows however, that not only the duration of the disease is influencing the RNFL thickness. Larger cohort studies are needed to identify further factors. Besides the RNFL the ganglion cell layer is known to be of great importance for LHON. Barducci et al. could even demonstrate that in the asymptomatic phase of the disease a GCL thinning could already be observed, underlining the importance of GCL in the early detection of LHON [17]. In our analysis we could again show as previous studies demonstrated that the GCL layer is thinner in chronic patients compared to acute LHON patients [28]. Interestingly, a study by Pajic et al. (2022) showed that GCL thickness is less reduced compared to other chronic atrophies of the optic nerve [29]. Other studies also analyzed the choroidal thickness and showed that a choroidal thinning in chronic LHON is strongly correlated with both RNFL and GCL thicknesses. These findings may suggest a pathophysiological mechanism involving vascular pathology of the choroid in relation to the retinal ganglion cell complex in LHON [18]. However, our retrospective study cohort did not allow for measurement of choroidal thickness but we implemented the measurement of macular EDI-OCT in our clinical routine to further evaluate this. In the future our results can be used for comparison of natural history OCT development and changes in patients receiving gene therapy. This could be beneficial for the therapy monitoring, also for other possible treatments.

### Multiple linear regression

Despite the significant thinning of GCL and RNFL in chronic compared to acute LHON patients, the correlation between the morphology and visual function was less pronounced. Though, we observed a significant correlation between RNFL and less GCL thickness with visual acuity, the high amount of unexplained variance suggests a more complex association. Still, RNFL and GCL thickness might be employed in cases of suspected aggravation and simulation of visual impairment. With help of multiple linear regression we aimed to enhance the model. However, multiple linear regression revealed only a significant correlation with RNFL and not with age, sex, mutation and time since onset. Certainly, these results are limited by the small sample size and larger cohorts should be used to retry a multiple regression. A very recent study analyzing 131 patients with different optical atrophies revealed that temporal GCL thickness shows the highest correlation with BCVA. However, this was not analyzed separately for the 11 LHON patients [30].

### Limitations

Our study cohort is of a retrospective nature and has consecutively the typical potential flaws of such analyses. Furthermore, the low sample size due to the rare incidence of the LHON could confound the results. Further prospective studies in a multicenter approach could proof our results and elucidate further the morphological changes during the course of LHON.

## Conclusion

We could show that there are significant differences between acute and chronic LHON patients visible in routine OCT scans. The retinal nerve fiber layer and ganglion cell layer thickness was significantly thinner in chronic patients to acute patients, showing a distinct sectorial pattern.

## Data Availability

The data that support the findings of this study are not openly available due to reasons of sensitivity and are available from the corresponding author upon reasonable request. Data are located in controlled access data storage Zenodo under the following link: https://zenodo.org/records/13908691?preview=1%25;26;token=eyJhbGciOiJIUzUxMiJ9.eyJpZCI6ImQ5MTFiMWIwLWVlMTgtNDRlZC05YTA2LTlmNDZiMjU0ZTJkZCIsImRhdGEiOnt9LCJyYW5kb20iOiIyZmFkNDE0YjdkM2ViNzE3ZWY0YzEwNDI1MWU2ZGNkOCJ9.lR4DPGHqXLDUdlOD40KeldUDnNcjZ_CGHzhY2YygRTwXniTqKwRSsMf4sKG4pxDzcHJrhszi-lnNmNYVIzBJGQ.
